# Lifestyle-dependent microglial plasticity: training the brain guardians

**DOI:** 10.1186/s13062-021-00297-4

**Published:** 2021-08-05

**Authors:** Marcus Augusto-Oliveira, Alexei Verkhratsky

**Affiliations:** 1 Laboratório de Farmacologia Molecular, Instituto de Ciências Biológicas, Universidade Federal Do Pará, Belém, 66075-110 Brazil; 2 Faculty of Biology, Medicine and Health, The University of Manchester, Manchester, M13 9PT UK; 3 Department of Stem Cell Biology, State Research Institute Centre for Innovative Medicine, 01102 Vilnius, Lithuania; 4 Achucarro Center for Neuroscience, IKERBASQUE, Basque Foundation for Science, 48011 Bilbao, Spain; 5 Department of Neurosciences, University of the Basque Country UPV/EHU and CIBERNED, Leioa, Spain

**Keywords:** Microglia, Neuroplasticity, Enriched environment, Physical exercise, Lifestyle modifications, Diet

## Abstract

Lifestyle is one of the most powerful instruments shaping mankind; the lifestyle includes many aspects of interactions with the environment, from nourishment and education to physical activity and quality of sleep. All these factors taken in complex affect neuroplasticity and define brain performance and cognitive longevity. In particular, physical exercise, exposure to enriched environment and dieting act through complex modifications of microglial cells, which change their phenotype and modulate their functional activity thus translating lifestyle events into remodelling of brain homoeostasis and reshaping neural networks ultimately enhancing neuroprotection and cognitive longevity.

## Introduction: lifestyle, neural plasticity and cognitive performance

Adaptive behaviours are paramount for survival: in complex multicellular organisms, environmental challenges instigate life-long morpho-functional restructuring of the nervous system, known as neural plasticity. Lifestyle is one of the most powerful instruments shaping mankind; the lifestyle includes many aspects of interactions with the environment, from nourishment and education to physical activity and quality of sleep. There is compelling evidence demonstrating that exposure of animals to environmental stimulation including enriched environment, social engagement and physical activity affects neural plasticity and impacts synaptic connectivity and neuronal morphology; similarly, dieting not only affects the organism as a whole but also reshapes structure and modifies functions of the nervous system.

In rodents engaged in physical activity (usually in a form of free access to the running wheel), an increase in the neuronal arborisation, length and complexity of dendrites, spine morphology and synaptic densities has been documented; these morphological changes develop in paralleled with an increased expression of glutamate receptors and amplification of long-term potentiation (LTP) in several brain regions [1–4]. This plastic remodelling seems to be associated with an increase in production of brain-derived neurotrophic factor (BDNF) [5]. The morpho-functional changes translate into improved cognitive performance including learning and memory [6, 7], prolong cognitive longevity [8–10] and reduce the risk of dementia [8, 11–13].

Intellectual engagement represents another lifestyle factor that, by instigating neural plasticity, positively impacts on cognitive longevity by increasing cognitive reserve. There is convincing evidence demonstrating the role of education, occupational activities, creativity challenges and social engagement in prolonging physiological cognitive ageing and delaying dementia [14–18]. Similarly, dieting has been shown to affect brain metabolism, neuronal plasticity, and synaptic connectivity [19–22] thus impacting of cognitive performance and cognitive longevity [23, 24].

Cellular mechanisms of lifestyle action on the brain remain to be fully elucidated; there is mounting evidence highlighting the role of neuroglia. Neuroglia are the principal homeostatic and defensive arm of the nervous system, which is critical for neural plasticity and cognitive performance. In particular, neuroglia are responsible for the ability of brain to compensate life-long pathological challenges thus preserving cognitive reserve [25]. Physical activity and enriched environment has been shown to significantly increase the complexity, volume and surface area of astrocytes, enhance astrocytic coverage of synapses and blood vessels and positively modulate astrocyte-dependent neurogenesis in adult neurogenic niches [25–29]. Dieting also affects astrocytes: for example, caloric restriction induces substantial increase in astrocytic complexity and increase in astrocytic synaptic coverage, which enhances control over extracellular glutamate and K^+^ thus augmenting long-term potentiation in the hippocampus of mice [30]. Astrocytes have been proposed to be a critical element in translating lifestyle factors into brain plasticity and cognitive capabilities [31]. Finally, diet, physical exercise, and environmental enrichment act on oligodendrocytes thus promoting myelination in physiology and pathology [32–35].

In this paper we shall overview the effects of lifestyle factors on the plasticity of the third major type of neuroglia— microglial cells, which contribute to brain physiology and represent the principal arm of the defence system of the central nervous system (CNS).

### Plasticity of microglia

**Fig. 1:**
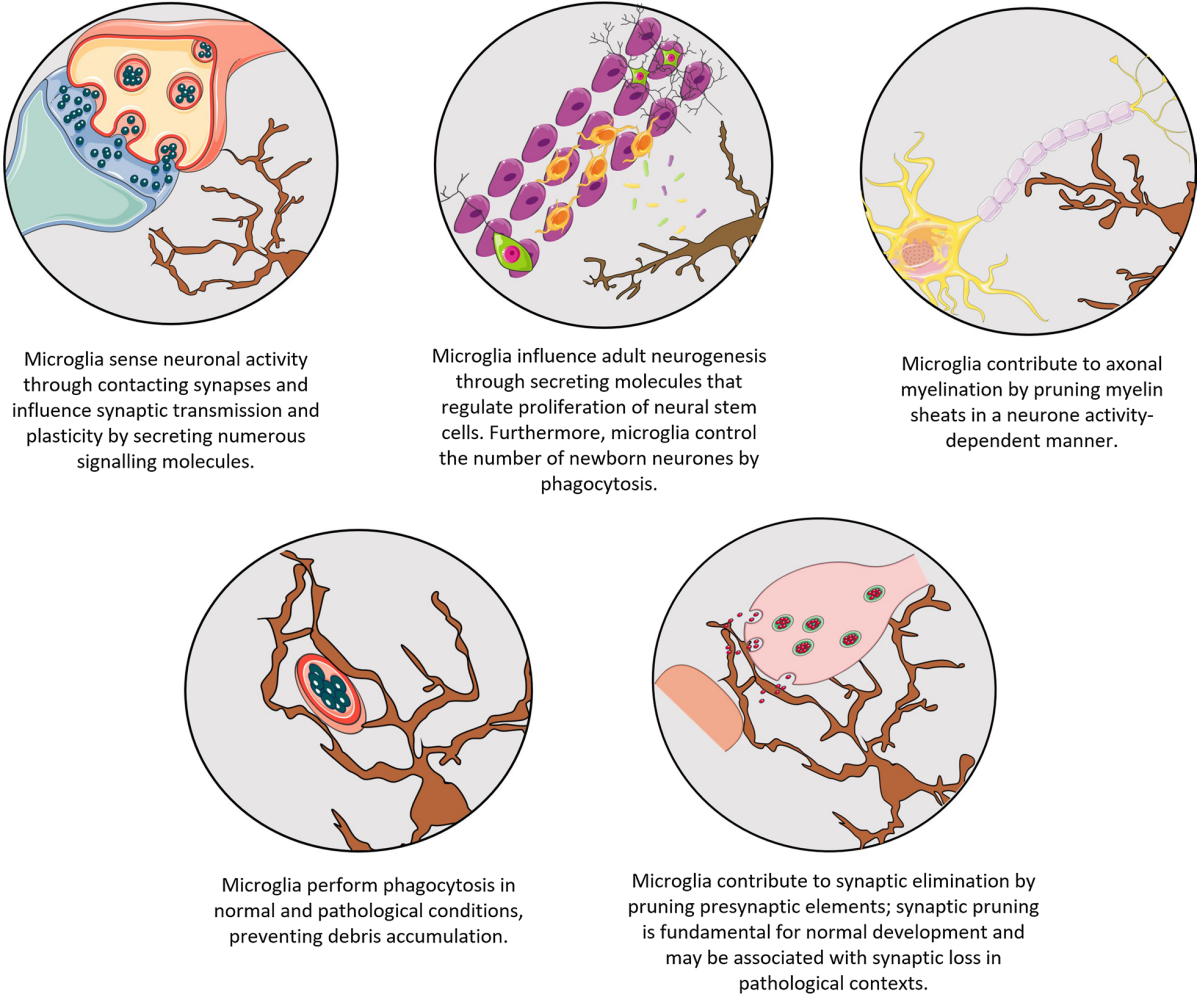
Microglial functions in physiology and pathophysiology

Microglia are the neural cells of the non-neural origin [36]: microglial precursors in the form of foetal macrophages invade the neural tube early in development [37, 38]. These precursors disseminate throughout the brain and the spinal cord and undergo the most remarkable metamorphosis. Mature microglial cells are as different from macrophages as they could be: the latter are spherical or amoeboid, while the former possess highly elaborated processes resembling, in their fundamental design, neural cells. The profound morphological transfiguration is accompanied by similarly profound physiological change: microglial cells acquire a multitude of receptors to neurotransmitters and neurohormones, while retaining "immune" receptors as a legacy from their myeloid ancestry, making microglia are, arguably, the most "sensitive" cells of the CNS. In the normal brain, microglia appear in the form of 'never resting' cells which constantly survey the nervous tissue by their highly ramified and moving processes; hence these cells are defined as 'surveilling microglia'. In addition, microglia perform numerous physiological functions related to regulation of synaptic behaviour, shaping synaptic contacts and regulating adult neurogenesis thus ultimately modulating cognitive processes (Fig. 1) [39–44]. Microglia are highly heterogeneous and plastic cells, which present numerous distinct morphological shapes and functional states depending on the brain region, age and context [45, 46].

### Lifestyle effects on microglia

#### Physical exercise

Physical exercise modifies density, morphological appearance, and molecular profile of microglia (Table 1). Ten days of physical exercise in the running wheel stimulates microglia proliferation in the superficial cortical layers [47], and favours ramified surveilling microglial state in the mouse hippocampus [48]. Microglia seem to translate numerous lifestyle modifications into changes in adult neurogenesis within neurogenic niches [49]. Physical exercises are known to stimulate neurogenesis, potentate survival of newborn neurones and improve memory [50–53]. Physical exercise induced rather profound changes in microglial phenotype, as these changes persisted even after cell isolation and maintenance in culture. Addition of purified (by FACS sorting of microglia isolated from transgenic Csf1r-GFP mice expressing GFP under control of *Csf1r* gene) microglia isolated from the brains of animals subjected to 3 weeks of voluntary running to the culture of hippocampal neurones obtained from sedentary mice activated neural cells and increased neurogenesis. These effects of microglia were mediated through colony-stimulating factor 1 (CSF-1) and its receptor signalling axis. Conversely, microglia from aged animals or young sedentary animals were not effective in recruiting and stimulating neuronal precursors [54]. The same CSF-1 signalling cascade underlies emergence of stress resilience following physical exercise [55]. Positive regulation of neurogenesis may also be mediated by an increase in microglial production of BDNF, which is well known enhancer of neurogenesis [56]. Voluntary physical activity increases the proportion of BDNF-expressing microglia in aged (but not in adult) mice microglia [57], while microglial levels of BDNF were found to correlate with the density of newly generated neurones. Physical activity also increases microglial production of pro-neurogenic insulin-like growth factor (IGF1), which may mediate local microglia-neural precursor cells communications [58]

**Table 1:** Effects of physical exercise on microglia

Species	CNS region	Experimental paradigm	Microglial changes	References
Mice	Whole brain	Treadmill for 6 weeks	In EAE model (transfer of encephalitogenic T cells), exercise protected the CNS against autoimmune inflammation by reducing microglial-derived ROS production, neurotoxicity and pro-inflammatory responses	[71]
Mice	Hippocampus or remaining brain	Running wheel for 10 weeks	Aged mice showed a greater proportion of CD86 and MHC II positive microglia. In aged females, access to a running wheel decreased proportion of CD86 and MHC II positive microglia in the hippocampus whereas aged males in the running group showed a decrease in the proportion of CD86 positive microglia in the brain and an increase in the proportion of MHC II positive microglia in hippocampus and brain	[63]
Mice	Hippocampus	Treadmill for 8 weeks	Treadmill running inhibited the excessive reactivity of microglia in hippocampus of the fluoride-toxic mice, accompanied with the alteration of neuroactive ligand-receptor interaction pathway	[140]
Mice	Spinal cord	Running wheel for 8 weeks	Exercise reduced microglial reactivity thus preventing age-related loss of motor neurones	[141]
Mice	Hippocampus	Treadmill for 9 days	Exercise protected against LPS-induced memory impairment with concomitant attenuation of IL-1β, TNF-α and IL-10 mRNA expression. Exercise abolished LPS-induced response of astrocytes and microglia	[59]
Rat	Hippocampus and striatum	Running wheel for 4 weeks	Exercise reduced microglial reactivity and partially prevented damage to dopaminergic neurones in a rat model of PD	[69]
Mice	Hippocampus	Running wheel for 2 weeks	Microglia mediate exercise-induced increase in neural precursor cell activity through fractalkine signalling	[54]
Mice	Hippocampus	Running wheel for 10 days	Exercise increases microglial proliferation without morphological, antigenic or transcriptional changes	[48]
Mice	Cortices	Running wheel for 10 days	Exercise led to regional increase in microglia proliferation	[47]
Mice	Striatum and Substantia nigra	Treadmill for 4 weeks	Exercise prevented dopaminergic neuronal loss by suppressing microglial reactivity in a PD model	[68]
Mice	Hippocampus	Treadmill for 12 weeks	Exercise preserved hippocampal cognitive function, suppressed β-amyloid accumulation in the hippocampus in APP/PS1 mice, and attenuates oxidative stress possibly through modulating microglia	[65]
Rat	Hippocampus	Treadmill for 4 weeks	Exercise inhibited reactive gliosis following STZ insult, reduced expression of pro-inflammatory mediators and enhanced expression of anti-inflammatory cytokine in the hippocampus	[142]

*EAE* Experimental autoimmune encephalomyelitis, *LPS* lipopolysaccharide, *APP/PS1* amyloid precursor protein/presenilin1 mouse Alzheimer's disease model mice, *PD* Parkinson disease, *ROS* reactive oxygen species

Although precise description of effects of physical exercise on microglia and mechanisms involved in physiological conditions needs more investigations, there is a large body of evidence indicating that physical exercise promotes microglia-dependent neuroprotection in numerous pathological contexts. For instance, physical exercise protects against lipopolysaccharide (LPS)-induced neuroinflammation and associated cognitive impairment. This protection is associated with suppressed expression of IL-1β, TNFα and IL-10 mRNA in the hippocampus, indicating reduced microglial pro-inflammatory response as an underlying mechanism by which physical exercise might protect CNS [59]. Similar mechanisms may be operational in ageing. Physical exercise, for example, reduces the ratio between pro- (IL-1β, IL-6 and TNFα) and anti-inflammatory (IL-10) cytokines in the hippocampus of aged rats [60]. Microglia are the main source of cytokines in the ageing brain [61], hence are likely to be responsible. Voluntary exposure to the running wheel for 8 weeks attenuated microglial proliferation in the hippocampus of aged mice [62], whereas running for 10 weeks reduced microglial reactivity in the hippocampus and other brain regions of aged rats [63]. Aged mice show greater expression of reactivity markers CD68 and MHCII; exposure of aged females to physical activity decreased densities of CD68 + and MHCII + microglia in the hippocampus, whereas in males CD68 + microglia decreased and MHCII + microglia increased in hippocampus [63]. These data suggest that the effects of physical exercise on microglial immunological profiles vary with age, sex and brain region, probably reflecting microglial heterogeneity. Treadmill exercise for 10 days attenuated cognitive decline and reduced glycolysis, glycolytic capacity, and PFKB3 enzyme in aged mice; similarly, senescent markers such as β-galactosidase and P16INK4A, were also reduced suggesting the exercise-related improved cognition is orchestrated by a normalisation of the metabolic profile and functionality of microglia [64].

Physical exercise-induced microglial plasticity contributes to neuroprotection in neurodegenerative diseases. In the APP/PS1 mice Alzheimer`s disease (AD) model, twelve weeks of treadmill exercise decreased β-amyloid deposits and improved cognitive processes possibly through hippocampal microglia modulation [65]. Treadmill exercise improved cognitive performance including spatial learning and exploratory activity, and reduced β-amyloid deposits and microglial reactivity [66]. In the Tg2576 mice AD model, three weeks of voluntary wheel running significantly reduced hippocampal levels of IL-1β and TNF-α and decreased soluble β-amyloid_40_ as well as soluble fibrillar β-amyloid [67]. These results indicate that physical exercise can shift the immune response in the brain of an AD mouse model by transforming microglia to an antigen presenting phenotype, thus reducing β-amyloid burden, alleviating AD pathology and improving cognition [67].

In a Parkinson`s disease (PD) MPTP mouse model, treadmill exercise for 4 weeks ameliorated dopaminergic neuronal loss by suppressing microglial reactivity, preventing loss of nigrostriatal neurones and improving motor balance and coordination dysfunction [68]. In 6-hydroxydopamine PD rat model running wheel exercise for four weeks suppressed microglia reactivity and partially prevented neuronal damage and cognitive decline [69]. This potent modulation of microglia, which have a significant neuroprotective role in the PD brain [70], highlights microglia as a key cellular element translating beneficial effects of physical exercise in PD.

Physical exercise and related microglial changes seem to protect the CNS in the experimental autoimmune encephalomyelitis model induced by transfer of encephalitogenic T-cells. High-intensity six-week continuous treadmill training reduced microglia reactive oxygen species formation, neurotoxicity and pro-inflammatory response, which are all involved in the propagation of autoimmune neuroinflammation [71].

#### Environmental enrichment

Environmental enrichment is defined as a brain stimulating environment composed by physical (such as puzzle boxes, toys, numerous feeders, ropes and running wheels) and social (relationships with peers) elements [72]. In humans, environmental enrichment corresponds to intellectual, social and physical engagement, which contributes to cognitive longevity [73]. Exposure to enriched environment results in well characterised beneficial effects on the CNS including boosting adult neurogenesis, synaptic plasticity, cellular physiology, and remodelling of neuroglia resulting in cognitive improvements and acceleration of neurological recovery following insults of various aetiology (Table 2) [31, 72, 74–77].

**Table 2:** Effects of enriched environment on microglia

Species	Brain region	Experimental paradigm	Microglial changes	References
Mice	Hippocampus, amygdala and hypothalamus	EE for 32 and 48 weeks	EE reduced expression of pro-inflammatory cytokines, increased Iba1 expression, and induced microglial hypertrophy and increased ramification	[79]
Mice	Hippocampus	EE for 7–8 weeks	EE prevents microgliosis induced by human β-amyloid oligomers, as evidenced by morphology, mRNA changes, and brain interstitial fluid cytokine levels	[81]
Mice	Hippocampus and hypothalamus	EE for 6 weeks	EE housing blocks pro-inflammatory cytokine gene induction and promotes arginase 1 mRNA expression in brain-sorted microglia, indicating that EE favours an anti-inflammatory activation state	[143]
Mice	Hippocampus and neocortex	EE for 6 weeks	EE in APP/PS1 mice amyloidosis model led to improved short-term memory, reduced microgliosis and increased microglial phagocytic activity	[83]
Mice		EE for 4–6 weeks	EE acting through enhanced β-adrenergic signalling reduces microgliosis in response to direct exposure to β-amyloid	[82]
Mice	Hippocampus	EE, PE, and EE + PE for 7 weeks	EE led to an increased microglial number at 5 and 10 months while PE and EE + PE increased microglial numbers only at 10 months	[78]
Mice	Amygdala	EE or PE for 40 days	EE Increased microglial proliferation	[144]
Rat	Hippocampus	EE for 12 weeks	EE ameliorates cognitive comorbidities associated with type I diabetes mellitus, possibly by reducing hyperactivity in the hypothalamic–pituitary–adrenal axis and microglial reactivity in diabetic animals	[91]
Mice	Hippocampus	EE for 87 weeks	Long-term EE reduces microglia morphological diversity of the molecular layer of dentate gyrus	[88]
Mice	Lateral septum	EE for 32 weeks	Following dengue infection, EE led to a reduction of microglial morphological diversity	[145]
Mice	Hippocampus, septum, olfactory bulb and brainstem	EE for 16 weeks	EE alleviated microgliosis, promoted faster viral clearance, decreased viral dissemination, reduced disease progression, and decreased CNS damage in a model of limbic encephalitis	[90]
Mice	Hippocampus	EE for 12 weeks	EE attenuated microgliosis, damage to the extracellular matrix and promoted virus clearance in a model of viral encephalitis	[89]
Mice	Striatum	EE for 7 weeks	Glioma-bearing mice housed in EE have increased branching and patrolling activity microglia, besides increased phagocytic activity	[92]
Pig	Frontal cortex	EE for 3 weeks	EE piglets displayed a signature consistent with a relative decrease in microglial activity compared to those in the standard condition	[146]

*EE* enriched environment, *PE* physical exercise, *APP/PS1* amyloid precursor protein/presenilin1 mouse Alzheimer's disease model mice

Environmental enrichment affects microglial densities. When 3-, 8- and 13-month-old C57BL/6 wild-type mice have been subjected to seven weeks of enriched environment, running wheel or combination of both, the density of Iba1 positive microglia in hippocampus increased. Enriched environment alone appeared to be more effective in increasing Iba1-labelled microglia; physical exercise on its own or in combination with environmental enrichment affected microglial density only in older animals. [78]. A longer environmental enrichment for 32–48 weeks promotes healthy ageing by reducing microglial expression of pro-inflammatory cytokines and MHCII. At the morphological level, environmental enrichment leads to microglial hypertrophy and increased ramification in hippocampus, hypothalamus and amygdala without changing microglial density [79]. In LPS-injection model of neuroinflammation, subjecting rats to enriched environment for 12 h/day for 7 weeks caused significant reduction of microglial reactivity with decreased expression of chemokines including Ccl2, Ccl3, Cxcl2, and cytokines including TNF-α and IL-1β [80].

Similarly, in the context of AD model, animals` exposure to the enriched environment boosts neuroprotection, which is, at least in part, associated with changes in microglia. In particular, exposure to enriched environment protected against direct β-amyloid toxicity through alleviating microglial reactivity, increasing microglial morphological complexity and decreasing expression of inflammatory cytokines such as IL-1β and TNF-α [81]. The underlying mechanism connecting environmental stimulation to the status of microglia is represented by an increased noradrenergic stimulation of the brain. This effect is mediated through activation of β-adrenoceptors: feeding mice with β-adrenergic agonist isoproterenol mimicked effects of environmental enrichment, whereas treating mice undergoing enriched environment with the β-adrenergic antagonist propranolol inhibited positive effect of environmental stimulation. This effect was also absent in transgenic animals lacking β_1,2_ adrenoceptors [82].

In the APP/PS1 mice AD model, environment enrichment for six weeks starting from 12 months of age, improved short-term memory, reduced microglial reactivity while increasing microglia phagocytic activity; the β-amyloid burden however remained unaffected [83]. Similarly, increased microglial phagocytic activity has been observed in 5xFAD AD mouse model subjected to six weeks of enriched environment as compared with animals in standard environment. In addition, exposure to environmental stimulation rescued adult neurogenesis and memory deficits simultaneously preventing β-amyloid dissemination [84]. An increase in microglial phagocytosis activity following exposure to enriched environment may also improve physiological ageing, known to suppress microglial phagocytic machinery [85].

Exposure to enriched environment does not always enhance microglial profiles. Systemic non-neurotropic dengue virus infection, for example, results in an increased size and complexity of microglia; exposure to the enriched environment reduced microglial diversity in lateral septum, with significant correlation between morphological complexity and the levels of TNF-α in the circulation [86]. At the same time, sedentary lifestyle negatively impacted on microglial reactivity, thus diminishing microglial neuroprotection [87]. Similar loss of morphological diversity occurs in the molecular layer of dentate gyrus in mice housed in long-term enriched environment, suggesting different microglial morphotypes may have different physiological roles in various environments, and that long-term enriched environment may be associated with adaptive microglial response to cognitive stimuli [88]. In Piry rhabdovirus model of encephalitis, mice exposed to enriched environment presented less CNS infection and substantially faster virus clearance, less microgliosis and less damage to the extracellular matrix than animals housed in standard environment [89]. In cocal virus infection, mice dwelling in standard environment demonstrated significant weight loss and higher mortality as compared with animals exposed to environmental stimulation. Additionally, enriched environment led to better locomotor and exploratory activity associated with less neuroinvasion and reduced microglial reactivity, revealing that enriched environment drives a more effective immune response in a mouse model of virus encephalitis [90].

In type 1 diabetic rats, enriched environment reduced microglial reactivity, improved memory and ameliorated cognitive comorbidities associated with diabetes [91]. Environmental stimulation also shapes microglial plasticity in glioma: glioma-bearing mice exposed to environmental stimulation have increased branching and patrolling activity of microglia, besides increased phagocytic activity [92].

#### Diet

Healthy diet, especially being applied in combination with other modifiable lifestyle factors discussed above, emerges as a promising strategy for preventing cognitive decline and promoting brain health [93, 94]. The adoption of a friendly diet or caloric restriction is positively associated with cognitive performance throughout lifespan, leading to cognitive improvements during infancy, adolescence and adulthood [95–97] and preserving cognitive functions in elderly [98]. The mechanisms by which diet affects the brain include modulation of synaptic plasticity, neuroglial support and adult neurogenesis [99]. On the other hand, high-fat diet is associated with obesity and cognitive impairments [100], which induces poor lifestyle choices leading to weight gain in a self-accelerating cycle [101].

Obesity is a world-wide concern which affects millions of people and represents an important risk factor for neurological disorders [102]. It is often caused by high-fat intake [103] and it is associated with impaired cognitive functions, neurodegenerative pathologies and atrophy in many brain areas including frontal lobe, anterior cingulate gyrus, hippocampus and hypothalamus [104]. Detrimental effects of high-fat diet on the brain include synaptic loss and microglial reactivity [105], the latter playing multiple roles in damaging the brain and affecting cognition [106–108]. On the other hand, the adoption of friendly low-fat diet rich in plant foods, moderate intake of fish, poultry and wine, and low intake of fat and red and processed meat promotes microglia-dependent neuroprotection through mitigating microglia-mediated brain disturbance [109].

Microglial appearance and functional activities are strongly affected by diet (Table 3); microglial changes contribute to brain response to both high-fat detrimental and low-fat friendly dieting. In Yucatan minipigs, for example, maternal high-fat diet during gestation and lactation modifies offspring's microglial density and morphology. These alterations occurred differently in hippocampus and prefrontal cortex; in the prefrontal cortex microglial density increased whereas in the hippocampus it remained unchanged compared to standard diet group; at a morphological level, anterior prefrontal cortex, dorsolateral prefrontal cortex and hippocampus presented higher number of unipolar microglia whereas orbitofrontal cortex presented higher number of multipolar microglia, both compared to standard diet group in hippocampus [110]. This brain region-dependent microglial response induced by high-fat diet is also observed in humans. Post-mortem analysis of the brain tissue obtained from obese individuals (body mass index, BMI, > 30) revealed increased microglial proliferation and morphological changes indicative of microglial reactivity (enlarged cell bodies and shortened processes) in the hypothalamus as compared with normal individuals (BMI < 25). At the same time in the cortex microglia kept physiological morphology (small cell bodies and ramified processes) in both obese and non-obese individuals [111].

**Table 3:** Effects of diet on microglia

Species	Brain region	Experimental paradigm	Microglial changes	References
Mice	Hypothalamus and total brain	LFD (6.5% fat), HFD (42% fat) and caloric restriction (40% less) for 6 and 24 months	HFD increased the number of microglia in the hypothalamus and both number and soma size of microglia were increased in the cerebellum during aging in HFD mice. Under basal- or LPS-induced inflammatory conditions, gene expression analysis of the total brain microglia population or hypothalamus tissue showed similar findings in HFD and LFD mice. Caloric restriction in LFD mice prevented the increased expression of phagocytic markers in white matter microglia with aging, and this protective effect of caloric restriction was not observed in HFD mice. Because running wheel access did not affect white matter microglia activation in either diet, dietary fat as well as caloric content may play an important role in the inflammatory process in brain aging	[123]
Mice	Hypothalamus	Standard diet (13,2% fat) or HFD (42% fat) for 28 days	HFD led to microglial reactivity and neuronal stress in the mediobasal hypothalamus. Microglial depletion abrogated HFD-induced hypothalamic inflammation besides to enhance leptin signalling and reduce food intake	[147]
Mice	Hippocampus and amygdala	HFD (60.3% fat) for 3 days	In the hippocampus, HFD induced enlarged synaptophysin boutons, indicative of neurodegeneration. In the amygdala, HFD exacerbated the effects of ageing on microglial priming (morphology) and significantly suppressed microglial phagocytosis	[115]
Mice	White matter	Western diet (42% fat)	WD diet induced an ageing-related metabolic dysfunction associated with impaired myelin-debris clearance in microglia, which is mediated by TGF-β signalling and disrupts lesion recovery after demyelination. Blocking TGF-β restores microglia responsiveness and myelin-debris clearance following demyelinating injury	[148]
Mice	Nucleus accumbens	high-caloric chocolate cafeteria diet for 43 days	This high-caloric diet led to microglial reactivity with increased expression of pro-inflammatory factors and abnormal responses after amphetamine-induced hyperlocomotion. Chronic inhibition of microglial reactivity normalised these behavioural alterations	[149]
Mice and Human	Hypothalamus	Mice: HFD (60% fat) for 8 weeks Human: post-mortem samples from obese individuals (BMI > 30)	HFD induced microglia number in the hypothalamus of mice. Gene expression analysis of isolated microglia found downregulation of genes important for sensing signals in microenvironment. In obese humans, it was found signs of hypothalamic gliosis and exacerbated microglial dystrophy	[111]
Mice	Hippocampus	HFD (60% fat) for 8 weeks	HFD partially disrupted the rhythmicity of circadian clock genes in microglia, besides disruption on microglial immune gene expression. HFD induced a shift of substrate utilisation on microglia, with decreased glutamate and glucose metabolism and an overall increase of lipid metabolism during active period of the animals	[150]
Mice	Hypothalamus	Caloric restriction (40% of the ad libitum food intake) in HFD and LFD animals for 23 months	Caloric restriction in combination with LFD affected microglial morphology and decreased expression of phagocytic markers (Mac2/Lgals3, Dectin-1/Clec7a and CD16/CD32 in microglia	[123]
Mice	Hippocampus	Luteolin intake (20 mg/d) for 4 weeks	In aged animals, luteolin food supplement improved spatial memory and restored expression of inflammatory markers compared with that of young animals	[131]
Rat	-	EO and EP intake (2%) for 8 weeks	In aged animals, this diet improved working memory. Then, blood serum was used to assess microglial response in vitro. BV-2 microglia treated with blood serum from EO- and EP-fed rat showed reduced expression of NO and TNF-α respectively	[133]
Mice	Hippocampus	HFD and LFD with or without blueberry (4%) for 5 months	HFD supplemented with blueberry had fewer microglia compared to LFD and HFD ones. BV-2 microglia treated with serum collected from mice fed the diets with blueberry produced less NO compared to HFD mice. HFD + blueberry mice presented higher levels of hippocampal BDNF and DCX-positive cells compared to mice fed HFD	[139]
Mice	Frontal cortex	Caloric restriction (70% of the ad libitum food intake) for 6 weeks and 6 and 12 months	Caloric restriction for 6- and 12 months counteracted ageing-induced microglial changes such as Ca^2+^ signalling and processes motility toward a younger phenotype. Even shot-term caloric restriction (6 weeks) beginning in old age significantly improved microglial motility and Ca^2+^ signalling	[124]

*LFD* low-fat diet, *HFD* high fat diet, *EO* Euterpe oleracea, *EP* Euterpe precatoria*,* *WD* Western diet

Distinct microglial response depends on the length of dieting. Hypothalamic microglia from mice fed with high-fat diet for 3 days upregulated expression of pro-inflammatory mediators including IL-1β, Cd74, Irf8 and IL-6. However, keeping the same mice on high-fat diet for eight weeks reduced expression of pro-inflammatory mediators and increased expression of anti-inflammatory molecules such as IL-10 and Pparg [111]. Early exposure (at postnatal days 21–60) of mice to a high-fat diet, triggered reactive microgliosis with increased expression of IL-1β and TNF-α, reduced neurogenesis and promoted immature morphology of dendritic spines along with reduced levels of scaffold protein Shank2 suggesting defective connectivity. In addition, these animals demonstrated cognitive impairment with spatial memory alterations [112]. Incubation of primary cultured microglia with palmitate, a saturated fatty acid present in high fat diet led to a secretion of exosomes which induced immature dendritic spine phenotype [112].

Similar changes were observed in mice fed with high-fat diet for eight weeks: animals showed reduced presence of synaptic markers, altered microglial morphology and cognitive disruption [105]. Recently, the mechanisms underlying obesity-associated cognitive decline were found to be influenced by reactive microglia. High-fat diet for 18 weeks made mice obese, which was associated with decreased dendritic spine density, increased microglial reactivity, increased microglial phagocytosis of synapses, which ultimately provoked cognitive impairment [106]. Reducing microglial reactivity with: (i) partial knockdown of fractalkine receptor CX3CR1; (ii) minocycline treatment, or (iii) annexin-V treatment prevented obesity-associated cognitive impairment. These data highlight microglial contribution to the synaptic loss and cognitive impairment associated with obesity [106].

In ageing, exposure to high-fat diet triggers reactive microgliosis in mice and in an APP/PS1 mouse model of amyloidosis [113]. Certain evidence indicates that even short-term consumption of high-fat diet may trigger cognitive deficits [114], although it remains doubtful whether this may be translated to humans. It has been suggested that the detrimental diet disrupts the ageing process by worsening the impact of ageing on microglial function and morphology, priming microglia in brain areas important for cognitive functions including hippocampus and amygdala [115, 116].

Appropriate diet therefore is critical for the brain performance and cognitive capabilities. In this context, diet-dependent modulation of microglia emerges as a non-pharmacological and non-invasive strategy to improve cognition and prolong cognitive longevity (Table 3) [117, 118]. Caloric restriction, in particular, protects the brain from age-dependent diseases and prolongs cognitive longevity [119–122]. Caloric restriction in combination with low-fat diet abolished age-dependent increase in Iba1 immunoreactivity, microglial density and expression of phagocytic marker Mac-2 in the white matter tract of hippocampus, the fimbria [123]. Caloric restriction for 6–12 months at 70% of *ad libitum* food intake has been shown to alleviate age-dependent dystrophic changes in microglia, manifested by decreased Ca^2+^ signalling and disorganised motility of microglial processes [124]. Combining the low-fat diet with caloric restriction reduced white matter microglial reactivity during ageing, modulating their morphology and reducing phagocytic markers [123]. Since microglial dystrophy is critical for ageing-induced brain dysfunction and cognitive decline, caloric restriction emerges as a non-pharmacological, cost-effective, and clinically relevant microglial modulator for rejuvenation of microglia.

Healthy diet with high content of flavonoids and phenolic compounds, present in plants, vegetables, and wine, protects cognition in subjects aged 65 or older. After 10 year`s follow up, individuals with highest flavonoids intake presented better cognitive performance compared with individuals with lowest intake: on average Mini-Mental State Examination score loss was 2.1 points in the latter, whereas in the former the score loss was only 1.2 points [125]. Polyphenol-rich diet is similarly associated with cognitive improvements in elderly [126]. Flavonoids and phenolic compounds are well known for their ability to affect microglial status, in particular by reducing microglial reactivity and restoring microglial homeostatic functions with beneficial influence on cognitive functions with consequent reduction in microglia-derived neuroinflammation and cognitive improvements [127–130]. Luteolin, a plant derived flavonoid, suppressed expression of pro-inflammatory genes in BV-2 microglia; whereas luteolin consumption significantly improved spatial working memory and reduced expression of inflammatory markers in hippocampi of aged (22–24 months old) mice [131]. Luteolin interacts with several signalling cascades modulating microglial transcriptomic profile and promoting anti-inflammatory and anti-oxidative phenotype, thus strengthening neuroprotection [132]. Similar outcomes were observed in ageing (19–21) months old rats fed with polyphenol rich açaí palm tree pulps; dietary supplementation with pulps of *Euterpe oleracea* (EO) and *Euterpe precatoria* (EP) for eight weeks improved working memory as tested by Morris water maze; the EO-supplemented diet, but not an EP one also improved reference memory. Treatment of BV-2 microglial cell line with serum obtained from rats receiving EO or EP rich diet reduced production of nitric oxide (NO) and expression of TNF-α [133].

Blueberries represent another source of polyphenols and anthocyanins well known to improve cognition especially in ageing [134–136], reduce microglial reactivity [137, 138], and counterbalance brain dysfunctions induced by high-fat diet [139]. In particular, mice exposed to the diet supplemented with blueberry showed significantly less Iba1 immunoreactivity and lower microglial density, together with higher levels of BDNF and larger number of newborn neurones. The BV-2 microglial cell lines treated with serum collected from mice fed with blueberry produced less NO [139].

## Conclusion: mens sana in corpore sana—microglia translate friendly lifestyle into brain health and cognitive longevity

**Fig. 2:**
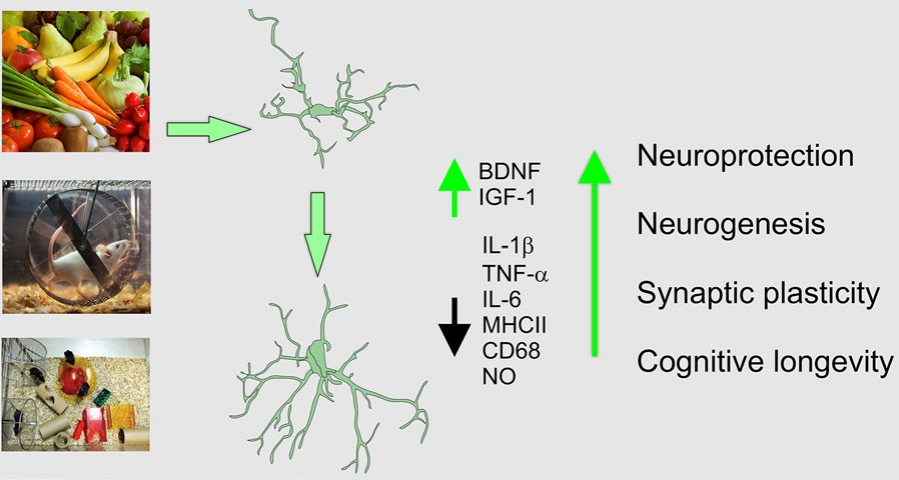
Microglia translate lifestyle into the neuroprotection and cognitive longevity. The adoption of friendly lifestyle induces morphological and functional plasticity of microglia, these plastic changes translate, at least in part, intellectual engagement, physical exercise and healthy diets into the brain health through enhanced neuroprotection, neurogenesis, and synaptic plasticity. Images of microglia has been re-drawn from ref [86] with permission

The brain is endowed with remarkable plastic capacity both in structure and cellular functioning, which allows life-long adaptation to the exposome. The adoption of healthy lifestyle including regular exercise, intellectual engagement and friendly diet significantly impacts the brain, affecting different areas at different levels of nervous tissue organisation modulating brain-wide homoeostatic systems such as blood–brain barrier and glymphatic clearance, remodelling cellular networks and modifying molecular cascades in neural and non-neural cells. As a holistic therapy, these lifestyle factors have been associated with improvements in cognitive performance, speedy recovery in the contexts of neurotrauma, stroke and neuroinfections, promoting healthy ageing and arresting or retarding neurodegenerative alterations, which as yet, cannot be managed pharmacologically. Lifestyle challenges, at least in part, are translated through changes in microglial phenotypes, that underlie multiple beneficial adjustments of the nervous tissue (Fig. 2).

## Data Availability

Not applicable.
